# Environmental Scenarios for the Future Nitrogen Policy in Flanders, Belgium

**DOI:** 10.1100/tsw.2001.289

**Published:** 2001-11-10

**Authors:** Stijn M.M. Overloop, Dirk E.L.J. Van Gijseghem, John F.M. Helming

**Affiliations:** Flemish Environmental Agency, Mechelen, Belgium

**Keywords:** nitrogen losses, manure policy, scenario analysis, nitrogen deposition, critical loads, ammonia, nitrous oxide, ex ante evaluation

## Abstract

The agricultural sector accounts for two thirds of nitrogen losses in Flanders, Belgium. Since 1991 both the government and the farmers have been taking measures to reduce the nitrogen surplus. Initially, the manure policy was aimed at distributing the manure surplus equally across Flanders. At the same time, the growth of livestock was stopped by a strict licensing policy, which required “command and control” measures. In recent years, the policy has switched to the use of individual target commitments by farmers. The Flemish manure policy will be tightened even more as a result of international pressures. An ex ante evaluation of possible policy options was carried out using three different scenarios spread out until 2010 (Business As Usual, Additional Measures, and Sustainable Development). To do this, a sector-economic, regionalized, environmental, comparative static, partial equilibrium, mathematical programming model of the Flemish agriculture was developed. The nitrogen emission into the agricultural soil was calculated by means of a regional soil balance. European targets can only be reached with manure processing, reduced fertilizer usage, and a strong reduction of intensive livestock breeding activities. The atmospheric deposition of nitrogen compounds will strongly decrease in 2010 if additional measures are taken. This will also result in a strong reduction of nitrous oxide emissions.

## INTRODUCTION

### Intensive Livestock Breeding in Flanders

Agriculture in Flanders is highly mixed. Soilless agricultural farms (intensive pig and poultry farming) exist alongside soil-dependent farms (dairy cattle and arable farming). Regional concentration has taken place and specialization has occurred mainly in soilless livestock farming. In 1999 the production value of Flemish agriculture amounted to 4.25 billion Euro and accounted for 72.9% of the total production value of Belgian agriculture. In 1998 agriculture accounted for 1.42% of Belgium's Gross National Product. The main sectors of Flemish agriculture are livestock and horticulture. Households spend 12.6% of their income on food products. In 1999 there were about 1.5 million cows, 7.3 million pigs, and 36 million poultry on 35,000 farms. These animals produce 212 million kg N, 50% coming from cows, 40% from pigs, and 10% from poultry. The agricultural surface of Flanders is 635,000 ha, of which 40% is grassland.

In 1998 manure surplus at farm level reached 60 million kg N, assuming maximum spreading according to the manure limits. One fourth of livestock farms had to deal with manure surplus purely based on the use of animal manure. Specialized pig breeding originates more than half (52%) of the farm surplus, and poultry breeding originates 10%.

In Europe the highest concentration of animals is found in the Netherlands and Flanders. Animal manure is very intensively applied to agricultural land. The manure surplus situation in certain regions of Flanders is due to an explosive growth of the livestock in the past 3 decades. Between 1965 and 1990, the number of pigs and poultry more than tripled. Between 1990 and 1998, the growth rate for pigs was 2% per annum and the growth rate for poultry was 5% per annum. Besides this growth, the cause of the surplus problem is the evolution from soil-dependent toward soilless specialized pig and poultry farms. Soilless agricultural farms were stimulated in the 1960s in order to intensify agricultural production. This has led to the concentration of intensive livestock farms close to producers of compound feed, which were usually located near rivers or close to harbors due to their dependence on imported feed stuff. Soilless livestock farms usually have contracts with feed producers. With this form of vertical integration, feed producers try to safeguard their market. In Flanders, approximately 60% of pig production and 90% of veal and poultry production takes place on a contractual basis between feed producers and farmers. The manure surplus problem is structurally linked with the agroindustrial development of the Flemish agriculture[1].

## Environmental Effects

In 1998 total nitrogen loss to the environment in Flanders equaled 245 million kg N. It consisted of losses to the air ($NO_x$, $N_2O$, $NH_3$: 123 million kg N), losses to the surface water (emissions from agriculture and wastewater treatment plants: 50 million kg N), and a residual compartment equal to 72 million kg N[2]. The latter comprised losses to soil, groundwater and nonestimated losses to surface water and into the air. The share of the economic sectors in the emissions of nitrogen and phosphorous amounted to 66% for agriculture, 20% for industry, 10% for population, and 4% for traffic. The agricultural sector accounted for 65% of the nitrogen emissions, which makes it the largest polluter of nitrogen. Agriculture accounted for 98% of the ammonia emissions, 49% of the nitrogen losses to surface water, and 50% of nitrous oxide emissions. Agriculture is 100% responsible for the residual compartment.

Losses to the surface water raise nitrogen concentrations. In 1999 the surface water quality met the legal basic quality standard for nitrate nitrogen (10 mg $NO_3$-N/l) at only 70% of the measuring points. The average concentration of nitrate nitrogen ($NO_3$-N) in surface water amounted to 5.2 mg N/l. In 1999 the ammonium nitrogen ($NH_4$-N) concentration met the Flemish basic quality standard of 5 mg $NH_4$-N/l at only 27% of the measuring points, with an annual average concentration of 3.5 mg $NH_4$-N/l. This illustrates clearly the relatively poor water quality in Flanders. Since 1999 a supplementary water quality monitoring network was set up to provide feedback to farmers on the effects of manure practices on surface water quality. Measured concentrations were evaluated against the standard of 11.3 mg $NO_3$-N/l (equals 50 mg $NO_3$/l), imposed by the European directive on nitrate (EC/91/676). This standard was exceeded at 60% of the measuring points at least once from July 1999 to June 2000. Peak values of 22 mg $NO_3$-N/l are regularly reached and exceeded.

Most (77%) of the atmospheric nitrogen deposition caused by air emissions in Flanders originates from agricultural activities. The share of reduced nitrogen (ammonia) in the atmospheric deposition amounts to 71%. In 1998 the average nitrogen deposition in Flanders was 39 kg/ha. However, more than 70 kg N/ha can be deposited locally. In 1998 the critical load for nutrient nitrogen was exceeded at 55% of 652 representative forest ecosystems. The value of the critical load varied from 7.5 to 13.6 kg N/ha[3].

Nitrogen losses from agriculture are calculated using the soil surface balance approach, according to the OECD methodology. Unlike the OECD method, ammonia emission is taken into account[4]. Fig. 1 illustrates the main streams of the soil surface

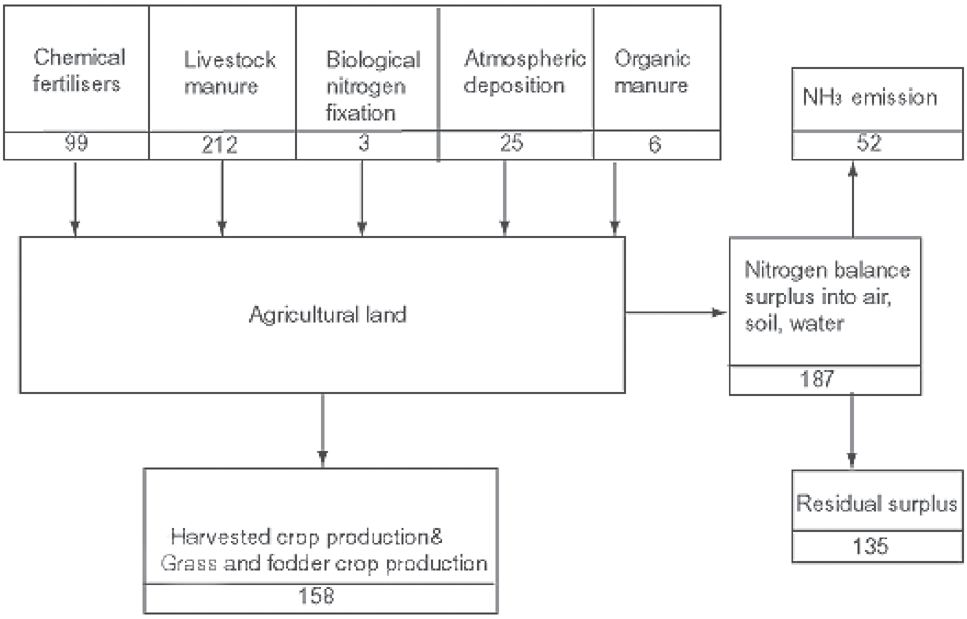

**FIGURE 1.** Soil surface balance of Flemish agriculture in 1998 in millions kg N[2].

balance of the Flemish agriculture. The input into the soil consists of livestock manure, chemical fertilizers, atmospheric deposition, organic fertilizers (compost, organic waste from the nutrition industry), and bacterial nitrogen fixation in the soil. On the output side, harvesting crops and grass and fodder crop production remove nutrients from the soil. Industrial manure processing and manure export are also considered as outputs, but were of no significance in 1998. The unbalance between input and output results in a nitrogen surplus and is lost into the environment. This surplus consists of ammonia emissions to the air and emissions to the soil. The latter can be broken down into the following streams: emissions to the air by denitrification processes, runoff to surface water, leaking to groundwater, and temporary stock in the soil.

In 1998, the nitrogen surplus amounted to 187 million kg N or 294 kg N/ha (inclusive ammonia emission), which equaled almost half of the inputs. The efficiency output/input in nitrogen use was 46%. A target value of 44 million kg N or 70 kg N/ha is set for the surplus, excluding ammonia emission. This is derived from the drinking water quality standard of 11.3 mg $NO_3$-N/l, imposed in the EU directive 91/676. The following assumptions have been made to derive the nitrogen surplus target: 50% of the nitrogen leaking out of the soil is denitrified during the migration through the soil; the annual rainfall surplus equals 300 mm. The European standard is already met at a leaking of 70 kg N/ha (3,000,000 l rainfall surplus/ha × 11.3 mg $NO_3$-N/l × 2 = 70 kg N/ha). If an equilibrium is assumed between nitrogen immobilization and mineralization, then a straightforward relationship exists between the soil surface nitrogen surplus and the concentration in ground- and surface water. The discrepancy to target needs to be minimized by 2003, otherwise the whole territory of Flanders would need to be designated as a vulnerable zone under pressure from the European Commission. This would lead to a restriction of the manure limits from an average of 225 to 170 kg animal N/ha. Table 1 shows a comparison of national soil surface nitrogen balances in the OECD and indicates that Flanders has a balance surplus even higher than the Netherlands.

**TABLE 1**
**Nitrogen Balance (in kg/ha) of Total Agricultural Land in 1995 to 1997[5]**

| | Nitrogen Balance |
|---|---|
| OECD | 23 |
| EU-15 | 58 |
| Belgium | 181 |
| Netherlands | 262 |
| U.S. | 31 |
| U.K. | 86 |
| France | 53 |
| Denmark | 118 |
| Germany | 61 |

## Policy Response

The manure policy in Flanders was designed to implement the Nitrate Directive (EC/91/676) of the European Community[5]. This directive on the protection of water from nitrate pollution from agricultural sources aims to reduce and prevent eutrophication problems of coastal and marine waters. The directive prescribes when and how the member states of the European Union should deal with the nitrate problems. It contains directives on how to treat livestock manure and chemical fertilizers in vulnerable regions.

The directive prescribes that member states need to:

1. Identify all surface waters and regions that are influenced or could be influenced by nitrate pollution. These regions are identified as regions where agriculture activities cause problems with drinking water quality or eutrophication.
2. Establish an action program for vulnerable regions. The measures stated in the directive are obligatory in these regions.
3. Set up a code of good agricultural practice to offer a basic protection level against pollution. This code should contain a minimum number of measures. Farmers need to be informed about this code.

The manure policy evolved in three phases. In the first phase (1991 to 1995), manure was seen as a resource, not as a waste, in order to reach the goals of the Nitrate Directive. The value of manure was recognized, primarily because of its nutrient content, but also because of its organic matter (farmyard manure). Manure rules were set up in order to avoid the application of manure in excess of the capacity of the environment. More severe area specific rules were required in order to protect some vulnerable areas. For farms with too little land to use the manure produced at the farm, the real solution consists of removing the surplus. Redistribution of nutrients is achieved by spreading slurry in the fields of neighboring farms or by transporting over longer distance. As the transportation costs were very high, a compulsory long distance transport system for larger farms was worked out. The targets of this first phase were reached: the transport of livestock manure from regions with surplus to regions where extra manure could be used was increased from 22 million kg N in 1992 to 60 million kg N in 1995. Consequently, distribution of manure from local livestock concentration areas to the whole territory of Flanders generated additional eutrophication.

During the second phase of the manure policy (1996 to 2000), restrictions were imposed at farm level. By means of a license policy, farmers were obliged to prove their past and future manure deposition and export (disposal). At the same time, the livestock sector was given until 2002 to reduce the manure surplus by means of source-oriented measures (forage techniques), effect-oriented alternatives (manure processing), or reduction of the livestock through natural or accelerated release of farmers. To compensate farmers for the negative socioeconomic consequences of this policy, family farms, which are considered to form the backbone of the Flemish agricultural sector, were positively discriminated.

Despite these measures, however, it remained impossible to spread all the manure on the available agricultural land. Therefore, in the third phase in 2000, a policy mix was worked out including the reduction of the number of animals, the use of feed concentrates with lower nutrient content, and manure processing (drying, burning, composting) or export of processed manure. The size of the livestock is kept at the 1995 to 1997 level until 2005 by controlling the manure volume produced at farm level.

An important innovation in this phase is the increased responsibility of the farmers for their manure practices. Farmers are allowed to use fertilizers beyond the manure limits if they can prove that the residual nitrate in the upper soil, which is 90 cm deep, is lower than 90 kg N/ha. This is measured during the period from October 1 to November 15. Farmers who obtain a better result than the nitrate residue regulation are rewarded. By 2002 additional scientific research will clarify whether the targets of the Nitrate Directive can be translated into a parcel-specific, controllable regulation and whether the current regulation is sufficient to prevent the eutrophication of the surface and groundwater. However, nitrogen is lost not only after harvest. Research in the Netherlands proved that the residual nitrate regulation is a highly uncertain policy instrument when the high cost, the need for additional control mechanisms by the government, and the low prediction value for the individual farmer are taken into account[6].

## MATERIALS AND METHODS

The third phase manure policy was evaluated in order to check whether the targets imposed by the Nitrate Directive could be reached on time. Also, additional measures were evaluated against the need to reach the targets after 2002. A brief description of applied methods and all results of this *ex ante* evaluation are published in the Environment and Nature Report Flanders: Scenarios MIRA-S 2000[1], which is a more comprehensive policy preparatory document. In order to evaluate the environmental policy of the agricultural sector a sector-economic, regionalized, environmental, comparative static, partial equilibrium, mathematical programming model of the Flemish agriculture was used, called Socio-Economic Agricultural Evaluation System (SELES), in combination with environment modules to calculate air emissions, use of water, energy, and pesticides[8]. SELES consists of two modules: a mathematical programming module called VRAM and an input-output module representing the Flemish economy called VLIO[9,10]. Only the results of the VRAM module are used to analyze the agricultural nitrogen balance. The mathematical programming module describes in detail all the options available to farmers in eight subregions of Flanders, called regional farms. The model is activity based and describes for every region nine livestock activity groups (milking cows, male beef cattle, female beef cattle, pigs, sows, laying hens, broiler, and broiler parents), two roughage activity groups (grassland and maize), nine arable activity groups (cereals, potatoes, sugar beets, cash crops with low nitrogen demand, cash crop with high nitrogen demand, dried vegetables, vegetables with low nitrogen demand, and a rest activity), and two vegetables in the open air groups (extensively grown and intensively grown). The activity “milking cows” is further split into four different technologies, representing observed differences in milk production per cow and nitrogen input per hectare on the farm. The fruit, horticulture under glass, and ornamental plant growing sectors are represented by each one aggregated activity. The rationale behind the selection of activities and technologies to describe the sectors is based on the potential contribution of these sectors to the manure problem in Flanders.

Regional balances for production and consumption within the model are included for intermediates: young stock, animal manure, and roughage. The levels of agricultural activities in VRAM are adjusted in such a way that each regional farm maximizes its profits while complying with constraints imposed by intermediate balances (manure, young stock, and roughage), primary input balances (availability of land and production quota), technical restrictions (mineral balances for animals, fertilization requirements) and environmental policies. The model shows in detail the consequences for regional production, nutrient surface soil balance, and regional farmers’ income. All input and output prices are exogenous, with exception of the intermediates. Mineral fertilizers and animal manure are modeled as substitutes into a fertilization balance, subject to an exogenous, crop-specific, minimum fertilization requirement. Input costs included in the objective function are purchased concentrates, chemicals, mineral fertilizers, and other inputs (hired labor, seed, etc.). Other elements of the cost function are application and industrial processing costs of animal manure, costs for imports and exports, and interregional transportation costs for tradable intermediates in Flanders. Assumptions for the price evolution are based on simulated effects of the European common agricultural policy rules (Agenda 2000) and farmers’ behavioral response to policy changes on a European level.The SELES results on livestock, land use, and animal manure production are used as inputs in the environmental modules. Outputs of these modules are calculated per unit of agricultural activity (animal or hectare). Ammonia emissions are calculated with generic emission coefficients for emission in pastures, in stables, and after manure application[11]. Calculations of nitrous oxide emissions are based on a methodology developed for the Intergovernmental Panel on Climate Change[12]. Nitrogen depositions are modeled using an atmospheric dispersion model[3].

The development of the environmental pressure from the agricultural sector is simulated, using four scenarios, starting in 1998 and tentatively using 2010 as the end date. The Autonomous scenario (AUT) is based on the economic development determined by growing markets and technological innovation, on the price policy of the European Common Agricultural Policy in the year 2000 and on the second phase manure policy. The Business As Usual scenario (BAU) incorporates the third-phase manure policy until 2003. The gradual tightening of manure limits restricts the size of the manure market. Livestock is maintained at the 1998 level until 2005. Manure processing is compulsory for large livestock farms (with more than 7500 kg phosphate production per year). A fixed processing cost per volume is used in the model. Animal manure must be spread out by low emission techniques such as subsoil injection, shallow injection into slots, or incorporation within 4 h. After 2004 licenses will be renewed on condition that the stables are ventilated with low emission techniques. In the Business As Usual Plus (BAU+) scenario, additional measures are added: tightening of the livestock manure limits until 170 kg N/ha from 2003, strong growth of organic farming (from 0.4% in 1998 to 10% in 2010, expressed in livestock numbers and land use), accelerated introduction of low emission stables, accelerated introduction of low nutrient forage, and the introduction of multiphase foddering for pigs. The last two measures partly neutralize the effect of lower manure limits. Manure processing is no longer compulsory. Additional measures in the Sustainable Development (SD) scenario reflect stronger ecological principles, giving organic farming a 25 to 50% share of total farming. The conversion to organic farming is assumed only for arable land activities, vegetable crops in open air, and milk production. Conversion costs are lowest in these activities and hence conversion is more feasible. Changes in the variable productivity, sales prices, and costs are dealt with by applying indices to the values for classic farming. These indices are a ratio of the considered variable for organic farming vs. classic farming. The time horizon of the SD scenario is of no realistic meaning, but the scenario is calculated against the background of 2005 (25% organic farming) and 2010 (50% organic farming). The SD scenario shows the effect of strong ecological measures. The outcome of the *ex ante* evaluation for the other economic sectors is taken into account for the modeling of the future atmospheric nitrogen deposition.

## RESULTS AND DISCUSSION

### Environmental Results

Model results include livestock evolution, which is the driving factor for the nitrogen surplus in Flanders. One of the driving factors in the evolution of cattle stock numbers is the European price policy. The beef cattle stock decreases by 85% in all scenarios in 2010 due to lower prices and exit from the constrained manure market. These results should be taken as an upper limit since subsidies by the Belgian government are not taken into account. Moreover, the model assumes that farmers easily shift between activities based on profitability. In the AUT scenario the manure production by cattle is substituted by the more profitable pig breeding activities (+17% in 2010). This substitution is observed in all scenarios, but the degree of substitution depends on the tightening of the manure policy. The total pig stock in the BAU, BAU+, and SD scenarios decreases from 1998 to 2010 by 3, 5, and 24% respectively. Installation and exploitation costs for additional measures in the BAU+ and SD scenarios are not taken into account in the model. These outcomes should therefore be considered as a minimum decrease. The poultry stock in 2010 increases by 7% in the AUT scenario, but decreases by 12% in the SD scenario. Poultry number is relatively stable in the BAU and BAU+ scenarios.

The surplus of the nitrogen soil surface balance decreases in the scenarios along with the tightening of the manure policy (Table 2). The driving force in the AUT scenario is the beef cattle stock reduction. The implementation of a manure limit 170 kg N/ha in the BAU+ scenario is not sufficient to reach the soil surface balance target. The surplus is only lower than the target value of 44 million kg N in the SD scenario. Chemical fertilizers are used less in all scenarios. The driving force here is the rising income of arable land farmers caused by increased acceptance of manure. Moreover, organic farming bans the use of chemical fertilizers. Biological nitrogen fixation is kept constant, although organic farming systems use relatively more leguminosae. Decreasing atmospheric depositions in the BAU and BAU+ scenarios are the result of planned or additional emission reduction measures in agriculture, industry, and traffic and transport. The nitrogen deposition decreases from 39 kg N/ha in 1998 to 29 kg N/ha in the BAU scenario and to 25 kg N/ha in the BAU+ scenario. This means that a deposition target of 26 kg N/ha is within reach in 2010. There is still a large discrepancy with ecological targets formulated as critical loads. In 2010 critical loads will be exceeded in 40% of the 652 selected forest ecosystems. To reach a zero excess, deposition should be lower than 14 kg N/ha. This is beyond the time horizon of this evaluation.

On the output side of the balance, plant production removes a decreasing amount of nitrogen through the decrease in fodder crop area, both across different scenarios and over time. Manure processing is only realized in the BAU and BAU+ scenarios. Facultative processing leads to lower volumes processed because

**TABLE 2**
**Soil Surface Balance for Nitrogen of the Flemish Agriculture in 1998 and for Four Scenarios (in million kg N)**

| | | | 2002 | | | 2010 | | | SD | |
|---|---|---|---|---|---|---|---|---|---|---|
| | | 1998 | AUT | BAU | BAU+ | AUT | BAU | BAU+ | 25% Organic | 50% Organic |
| Input | Livestock manure | 212 | 205 | 181 | 149 | 175 | 157 | 125 | 115 | 115 |
| | Chemical fertilizers | 99 | 103 | 83 | 79 | 109 | 89 | 93 | 70 | 51 |
| | Organic manure | 6 | 6 | 6 | 6 | 6 | 6 | 6 | 6 | 6 |
| | Atmospheric deposition | 25 | 25 | 21 | 19 | 23 | 18 | 16 | 14 | 14 |
| | Nitrogen fixation | 3 | 3 | 3 | 3 | 3 | 3 | 3 | 3 | 3 |
| Output | Crop production | 158 | 154 | 150 | 147 | 145 | 144 | 140 | 137 | 141 |
| | Manure processing and export | 0 | 0 | 6 | 0 | 0 | 13 | 9 | 0 | 0 |
| Surplus | Ammonia emission | 52 | 53 | 38 | 31 | 49 | 35 | 27 | 23 | 23 |
| | Residual surplus | 135 | 135 | 100 | 78 | 122 | 82 | 67 | 48 | 25 |
| | Residual surplus in kg N/ha | 213 | 212 | 157 | 123 | 192 | 129 | 105 | 76 | 39 |

of the relatively high processing costs. All processed manure is for export.

The ammonia emission, which is part of the surplus, is decreasing both across all scenarios and over time, thanks to lower manure production, manure processing, and low emission techniques. The Flemish target is set at 37 million kg N for all sectors and is derived from the national emission ceilings, agreed at the Göteborg protocol of the Long Range Transboundary Air Pollution protocol of the United Nations of December 1, 1999. Hence the target can be reached easily, since agriculture produces 98% of the emission.

An indirect consequence of the manure policy is the important reduction of nitrous oxide emission in all scenarios. This illustrates the integrated effect of this policy. In 2010 reductions are up to 11% in the AUT scenario and 44% in the SD scenario compared to 1998 when 8 million kg N were emitted as nitrous oxide. This reduction is the effect of lower livestock manure production and lower use of chemical fertilizers. The effect of low emission spreading techniques on subsoil denitrification is not accounted for. In comparison with other economic sectors, agriculture has a greater potential for emission reduction. On the contrary, despite a gain in efficiency, all other sectors show an increase in emission due to a volume increase.

### Discussion of the Model

In this research, a regionalized sector model is used to evaluate the possible impact of manure and ammonia policies on the agricultural sector in Flanders. The model has both advantages and disadvantages for the economic problem at hand[13]. An important advantage of the model is that the agricultural sector is described as a whole. This is important because, through the manure and land balances, nitrogen policies not only affect the producers, but also the users of animal manure. A disadvantage is that activities are aggregated to the regional level in order to keep the model and the computation time manageable. One of the consequences of this aggregation is that manure transportation costs within regions are not taken into account. It could be argued that these costs are small compared to application costs and transportation costs between regions. Another consequence is that behavioral and structural differences between farms are not taken into account. Farm models would be more suitable to do this. They would also be more suitable to include more technical options than the ones modeled at the aggregate level. However, changes in aggregate demand and supply and resulting changes of market prices cannot be modeled by farm models. The effects of nitrogen policies go beyond the individual farm and therefore at least the impact on manure and land prices should be taken into account[13]. This requires more aggregate models to model effects of important policy changes. Another weakness is that the model focuses on profit maximization by the individual farmer. In reality, the farmers' behavior is more diverse, which explains a different response to policies than the one predicted by the model. It should be noted that the model should not be used as a tool to predict the future, but as a decision support system to choose between different policy options.

## CONCLUSIONS

The *ex ante* evaluation of nitrogen policy in agriculture shows that the nitrogen surplus can only be reduced by drastic measures. The current and planned policy (BAU scenario) will not be sufficient. Assuming that manure processing will be restricted in application through high costs, livestock reduction and more efficient use of animal manure become necessary. In the SD scenario this is realized by conversion to organic farming on a large scale. This implies a conversion to extensive (low input/ha) farming, both on arable land and in milk production. Conversion to extensive farming could be realized by subsidies for livestock reduction. This policy instrument would be very expensive for an effective reduction. The effects of nitrogen pollution on vulnerable nature were evaluated for deposition effects, but measures equivalent to the ones implemented in the SD scenario are required as a minimum in order to close the gap to the long-term target. Finally, the manure policy has a positive side effect on the climate change policy through the reduction of nitrous oxide emissions.

## References

1. Van Gijseghem, D. (1997) Implementation of nitrate policies in Flanders. In *The Implementation of Nitrate Policies in Europe: Processes of Change in Environmental Policy and Agriculture*. Brouwer, F. and Kleinhanss, W., Eds. Wissenschaftsverlag Vauk, Kiel, KG. pp. 135–147.
2. Van Steertegem, M., Ed. (2000) *Milieu- en Natuurrapport Vlaanderen: scenario's MIRA-S 2000*. Garant, Leuven/Apeldoorn (in Dutch; English summary).
3. Mensink, C., Janssen, L., and Overloop, S. (2001) A GIS based modelling system for the assessment of critical load exceedances in forest ecosystems. In *Ecosystems and Sustainable Development III*. Villacampa, Y., Brebbia, C.A., and Uso, J.L., Eds. WIT Press, Southampton, U.K. pp. 195–204.
4. Parris, K. (1998) Agricultural nutrient balances as agri-environmental indicators: an OECD perspective. In *Proceedings of the First International Nitrogen Conference*. van der Hoek, K.W., Erisman, J.W., Smeulders, S., Wisniewski, J.R., and Wisniewski, J., Eds. Elsevier, Amsterdam. pp. 219–226.
5. OECD. (2001) Environmental indicators for agriculture, volume 3, methods and results. Paris.
6. Goodschild, R.G. (1998) EU policies for the reduction of nitrogen in water: the example of the Nitrate Directive. In *Proceedings of the First International Nitrogen Conference*. van der Hoek, K.W., Erisman, J.W., Smeulders, S., Wisniewski, J.R. and Wisniewski, J., Eds. Elsevier, Amsterdam. pp. 737–740.
7. Corré, W. (1994) Bepaling van de hoeveelheid minerale stikstof in de bodem in het najaar als instrument voor het te voeren stikstofbeleid. Rapport 21, DLO-IAB. Haren (in Dutch).
8. Helming, J.F.M., van Bruchem, C., Geertjes, K., van Leeuwen, M.G.A., Veenendaal, P.J.J., van Gijseghem, D., and Overloop, S. (2001) Milieugevolgen van de landbouw in Vlaanderen, 1991–2010. Report 3.01.02, LEI. The Hague (in Dutch; English summary).

9. Helming, J.F.M., Peeters, L., and Veenendaal, P.J.J. (2001) Assessing the consequences of environmental policy scenarios in Flemish agriculture. In *Agricultural Sector Modelling and Policy Information Systems*. Hackelei, T., Witzke, H.P, and Henrichsmeyer, W., Eds. Proceedings of the 65th European Seminar of the European Association of Agricultural Economists (EAAE). Wissenschaftsverlag Vauk, Kiel, KG. pp. 237–245.
10. Reyns, L., Peeters, L., and Veenendaal, P.J.J. (1999) Manure in Flanders: assessing the economic consequences of alternative abatement policies. In *Agriculture and the Environment: Challenges and Conflicts for the New Millenium.* Turner, S.T.D. and Alford, D. Eds. ADAS, Wolverhampton. pp. 93–100.
11. Pollet, I. (1996) Onderzoeks- en ontwikkelingsovereenkomst inzake de NH3-emissie door de landbouw. Rapport 174M3495, Ghent University, Ghent (in Dutch).
12. Van Moortel, E., Boeckx, P., and Van Cleemput, O. (2000) Inventory of nitrous oxide emissions from agriculture in Belgium—calculations according to the revised 1996 Intergovernmental Panel on Climate Change guidelines. *Biol. Fert. Soils* **30**, 500–509.
13. Helming, J.F.M. (1998) Effects of nitrogen input and nitrogen surplus taxes in Dutch agriculture. *Cah. Econ. Sociol. Rurales* **49**, 5–31.

| | | |
|---|---|---|
| Received: | July | 16, 2001 |
| Revised: | October | 3, 2001 |
| Accepted: | October | 9, 2001 |
| Published: | November | 10, 2001 |
